# Inhibition of phosphodiesterase-5 suppresses calcineurin/NFAT- mediated TRPC6 expression in pulmonary artery smooth muscle cells

**DOI:** 10.1038/s41598-017-06350-5

**Published:** 2017-07-20

**Authors:** Shaojun Li, Yilin Pan, Rui Ke, Xinming Xie, Cui Zhai, Wenhua Shi, Jian Wang, Xin Yan, Limin Chai, Qingting Wang, Qianqian Zhang, Xiaofan Su, Lan Yang, Li Gao, Manxiang Li

**Affiliations:** 1 Department of Respiratory and Critical Care Medicine, The First Affiliated Hospital of Xi’an Jiaotong University, Xi’an, Shaanxi 710061 P.R. China; 2 Division of Allergy and Clinical Immunology, Department of Medicine, The Johns Hopkins University School of Medicine, Baltimore, MD 21224 USA

## Abstract

The up-regulation of transient receptor potential channel 6 (TRPC6) has been found to contribute to the proliferation of pulmonary artery smooth muscle cells (PASMCs), and inhibition of phosphodiesterase-5 (PDE5) has been shown to suppress TRPC6 expression in PASMCs. However, the molecular mechanisms underlying the up-regulation of TRPC6 expression and PDE5 modulation of TRPC6 expression in PASMCs remain largely unclear. The aim of this study is to address these issues. Endothelin-1 (ET-1) dose and time-dependently up-regulated TRPC6 expression in primary cultured rat PASMCs, and this was accompanied with the activation of calcineurin and subsequent translocation of NFATc4 to the nucleus. Further study indicated that inhibition of calcineurin by cyclosporine A or knockdown of NFATc4 using small interfering RNA suppressed ET-1-induced TRPC6 up-regulation. In addition, luciferase reporter assay showed that NFATc4 directly regulated the expression of TRPC6 in PASMCs. Inhibition of PDE5 by sildenafil suppressed ET-1-induced activation of calcineurin/NFATc4 signaling pathway and consequent TRPC6 up-regulation in PASMCs, while these inhibitory effects of sildenafil were abolished by PKG inhibitor Rp-8Br-cGMPs. Taken together, our study indicates that ET-1 stimulates TRPC6 expression by activation of calcineurin/NFATc4 signaling pathway, and inhibition of PDE5 suppresses calcineurin/NFATc4- mediated TRPC6 expression in PASMCs in a cGMP-PKG-dependent manner.

## Introduction

Pulmonary hypertension (PH) is a life-threatening disease of various origins characterized by severe remodeling of the pulmonary vascular that leads to increased pulmonary vascular resistance and pulmonary arterial pressure, ultimately resulting in right heart failure and premature death^1, 2^. Pathologic changes leading to vascular remodeling include endothelial dysfunction, increased migration/proliferation of pulmonary artery smooth muscle cells (PASMCs) and fibroblast proliferation, and abnormal deposition of extracellular matrix^3^. Increased proliferation of PASMCs is the most prominent feature of pulmonary vascular remodeling^4^. Therefore, it is essential to explore the molecular mechanisms associated with the proliferation of PASMCs to ameliorate pulmonary vascular remodeling and thus to treat PH.

The calcium ion (Ca^2+^) is a fundamentally important second messenger that regulates numerous cellular processes such as cell migration, proliferation, differentiation, contraction and apoptosis in a wide variety of cell types^5, 6^. The elevation in intracellular calcium concentration ([Ca^2+^]i) in PASMCs is a major trigger for pulmonary vasoconstriction, as well as a critical stimulus for cell proliferation and migration^7^. [Ca^2+^]i in PASMCs can be increased by Ca^2+^ release from intracellular stores and Ca^2+^ influx through Ca^2+^-permeable cation channels in the plasma membrane. The canonical transient receptor potential channels (TRPCs) are non-selective Ca^2+^-permeable cation channels that are widely expressed among tissues with different functions. Among the seven known isoforms, TRPC6 is an important isoform highly expressed in PASMCs and plays an important role in the pathogenesis of PH^8–10^. TRPC6 expression has been found to be up-regulated in PASMCs from patients with idiopathic pulmonary arterial hypertension (IPAH)^11^. Furthermore, the up-regulation of TRPC6 has been shown to contribute to mitogen-mediated PASMCs proliferation^12, 13^.

Endothelin-1 (ET-1), a 21-amino acid mammalian peptide mainly synthesized by vascular endothelial cells, is a powerful vasoconstrictor and mitogen for PASMCs^14^. Plasma level of ET-1 has been found to be significantly elevated in patients and animals with PH^15, 16^. Furthermore, the mitogenic effect of ET-1 on PASMCs has been confirmed to play a prominent role in pulmonary vascular remodeling in PH^17^. Activation of several pro-proliferation signaling pathways has been found to be responsible for ET-1-induced PASMCs proliferation^18–21^. Study by Kunichika *et al*. has demonstrated that TRPC6 up-regulation is involved in ET-1-mediated PASMCs proliferation^13^. However, the detailed molecular mechanisms underlying TRPC6 up-regulation induced by ET-1 in PASMCs are still unclear.

Sildenafil is a potent and highly selective phosphodiesterase-5 (PDE5) inhibitor used as a major agent for the clinical treatment of PH^22^. Recently, it has been found that sildenafil can inhibit TRPC6 expression in PASMCs, thereby contributing to the therapeutic effects on chronically hypoxic pulmonary hypertension (CHPH) in rats^23, 24^. However, whether inhibition of PDE5 by sildenafil also modulates the expression of TRPC6 induced by ET-1 in PASMCs and what are the possible mechanisms for that are still unknown. To explore the mechanisms of TRPC6 up-regulation induced by ET-1 in PASMCs, and to examine the effect of PDE5 inhibition on ET-1-induced TRPC6 expression and its potential mechanisms, primary cultured PASMCs were stimulated with ET-1, TRPC6 expression and activation of the calcineurin/NFAT signaling pathway were determined, then the effects of sildenafil on these changes and the underlying molecular mechanisms were further investigated.

## Results

### ET-1 increases TRPC6 expression in rat PASMCs

**Figure 1:**
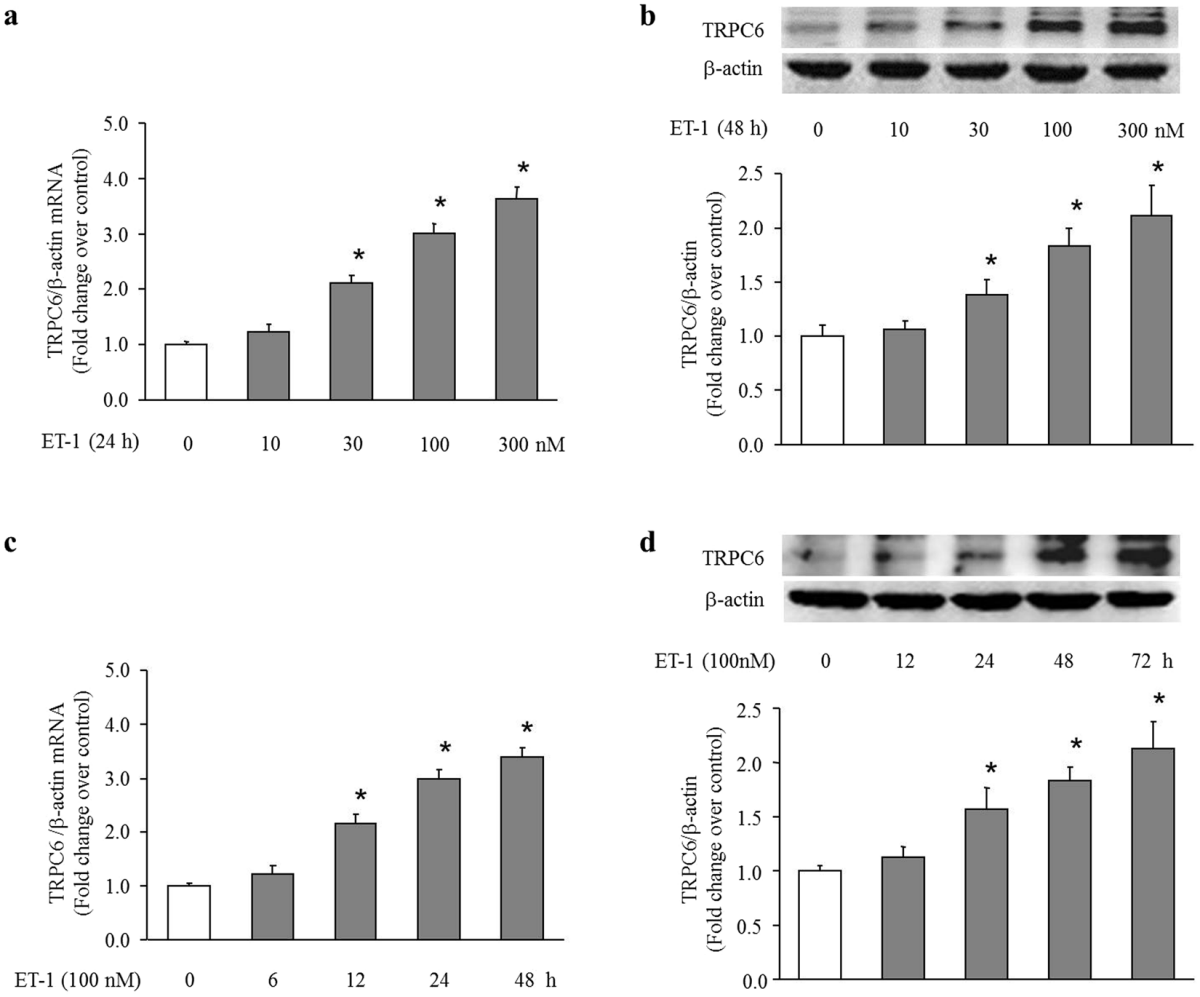
ET-1 dose and time-dependently increases TRPC6 expression in PASMCs. (**a**) PASMCs were stimulated with different concentration of ET-1 ranging from 0 to 300 nM for 24 h, TRPC6 mRNA level was determined using qRT-PCR, β-actin served as an internal control (n = 4 each group). (**b**) PASMCs were stimulated with different concentration of ET-1 ranging from 0 to 300 nM for 48 h, TRPC6 protein level was determined using immunoblotting, β-actin served as a loading control (n = 4 each group). (**c**) PASMCs were exposed to 100 nM ET-1 for the indicated times. TRPC6 mRNA level was determined using qRT-PCR, β-actin served as an internal control (n = 4 each group). (**d**) PASMCs were exposed to 100 nM ET-1 for the indicated times. TRPC6 protein level was determined using immunoblotting, β-actin served as a loading control (n = 4 each group). *P < 0.05 versus control.

To examine whether ET-1 induces TRPC6 up-regulation in PASMCs, cells were treated with different concentrations of ET-1 for different times, the expression of TRPC6 was determined using qRT-PCR and Western blotting. As shown in Fig. 1a and b, ET-1 dose-dependently increased TRPC6 expression in PASMCs, 100 nM ET-1 caused a 2.99-fold increase in TRPC6 mRNA level at 24 h compared with control, and a 1.83-fold increase in TRPC6 protein level at 48 h compared with control (both P < 0.05). Figure 1c demonstrates that 100 nM ET-1 up-regulated TRPC6 mRNA expression in a time-dependent manner after 12 h treatment in PASMCs, TRPC6 mRNA level raised to 3.37-fold over control at 100 nM ET-1 for 48 h incubation (P < 0.05). Figure 1d shows the time course of 100 nM ET-1 regulation of TRPC6 protein level, which increased to 2.13-fold over control at the time of 72 h (P < 0.05). These results suggest that ET-1 increases TRPC6 expression in PASMCs.

### ET-1 induces activation of calcineurin/NFATc4 signaling pathway

**Figure 2:**
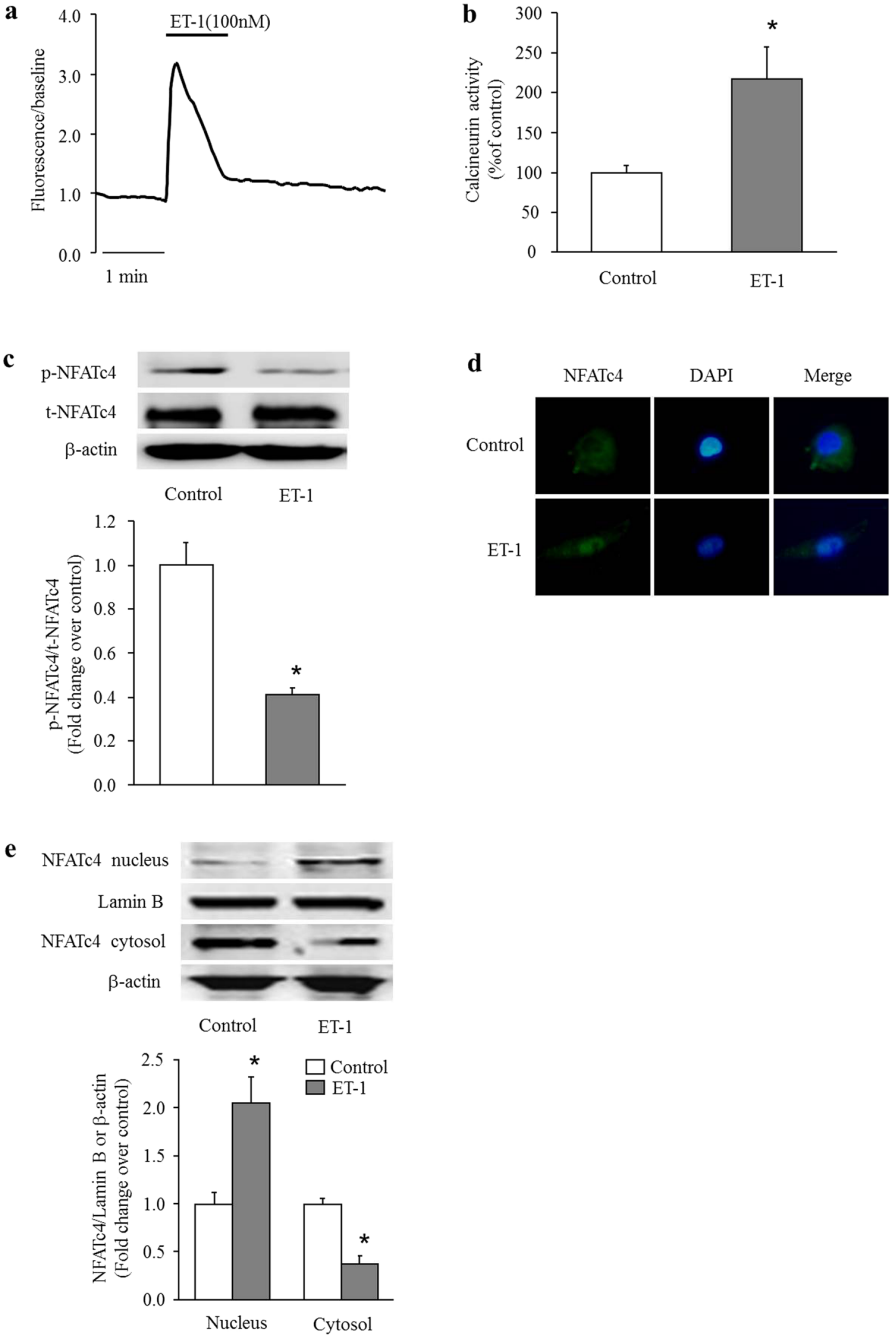
ET-1 induces activation of calcineurin/NFATc4 signaling pathway. (**a**) Representative traces demonstrating the effect of ET-1 (100 nM) on intracellular calcium concentration ([Ca^2+^]i) in PASMCs. (**b**) PASMCs were stimulated with 100 nM ET-1 for 30 min, calcineurin activity was determined using a calcineurin assay kit (n = 4 each group). (**c**) PASMCs were incubated with 100 nM ET-1 for 3 h, phosphorylation of NFATc4 was measured by immunoblotting, β-actin served as a loading control (n = 4 each group). (**d**) PASMCs were incubated with 100 nM ET-1 for 3 h, subcellular localization of NFATc4 was determined using immunofluorescence staining. Cells were stained with a primary antibody for NFATc4 and an Alexa Fluor 488-conjugated secondary antibody. Cell nuclei were stained with DAPI. (**e**) PASMCs were incubated with 100 nM ET-1 for 3 h, protein level of NFATc4 in the cytoplasmic and nuclear fractions were determined with immunoblotting. Lamin B and β-actin served as loading controls for the nuclear and cytoplasmic fractions, respectively (n = 4 each group). *P < 0.05 versus control.

Calcineurin is a calcium-calmodulin-dependent serine/threonine protein phosphatase. Activated calcineurin dephosphorylates nuclear factors of activated T cells (NFAT), which then translocate to the cell nucleus, where they can regulate transcription of NFAT-responsive genes, including TRPC6^25–27^. Here, we examined whether ET-1 elevates intracellular calcium concentration, subsequently triggers calcineurin/NFAT signaling pathway activation in PASMCs. Figure 2a shows that ET-1 (100 nM) caused a significant increase in [Ca^2+^]i in PASMCs. Figure 2b demonstrates that treatment of PASMCs with ET-1 (100 nM) for 30 min induced a 2.17-fold increase in calcineurin activity compared with control (P < 0.05). We next examined the effects of ET-1 on the phosphorylation and distribution of NFATc4 in PASMCs. As shown in Fig. 2c, treatment of PASMCs with ET-1 (100 nM) for 3 h significantly reduced the phosphorylation level of NFATc4 without affecting the total level of NFATc4, the phosphorylation level of NFATc4 declined to 0.41-fold over control (P < 0.05). As expected, fluorescence-labeled NFATc4 was predominantly located in the cytoplasm in control cells, while after treatment with ET-1 (100 nM) for 3 h, fluorescence-labeled NFATc4 was significantly increased in the nucleus (Fig. 2d). Figure 2e shows that ET-1 (100 nM) significantly increased NFATc4 protein nucleus translocation after 3 h treatment, which increased to 2.05-fold over control (P < 0.05). These results suggest that ET-1 effectively activates the calcineurin/NFATc4 signaling pathway in PASMCs.

### Activation of the calcineurin/NFATc4 pathway mediates the effects of ET-1 on TRPC6 expression in PASMCs

**Figure 3:**
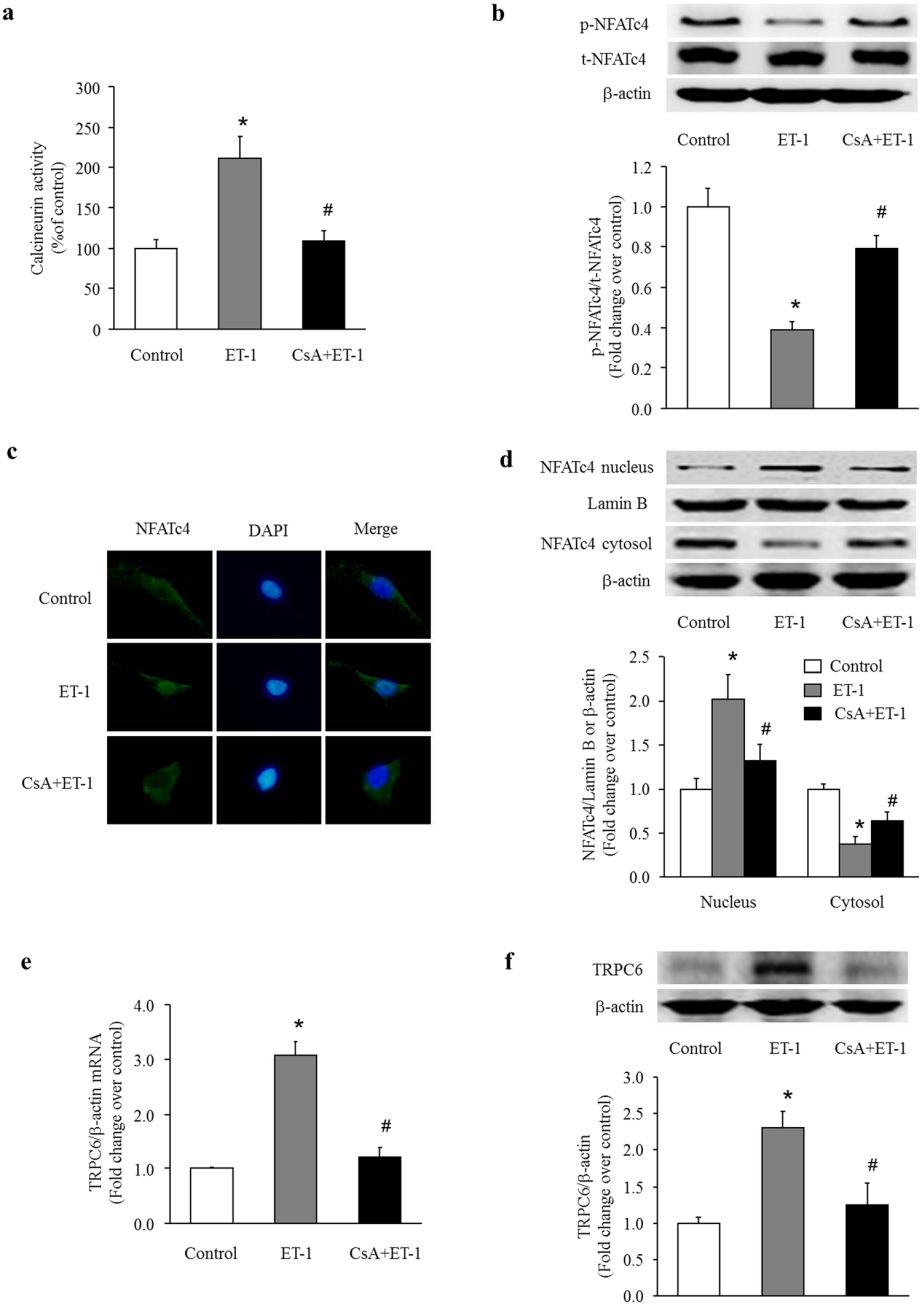
Activation of calcineurin mediates the effects of ET-1 on TRPC6 expression in PASMCs. (**a**) PASMCs were pre-treated with or without calcineurin inhibitor cyclosporine A (CsA) (10 μM) for 30 min before stimulation with ET-1 (100 nM) for 30 min, calcineurin activity was determined using a calcineurin assay kit (n = 4 each group). (**b**) PASMCs were pre-treated with or without CsA (10 μM) for 30 min before stimulation with ET-1 (100 nM) for 3 h, phosphorylation of NFATc4 was measured by immunoblotting, β-actin served as a loading control (n = 4 each group). (**c**) PASMCs were pre-treated with or without CsA (10 μM) for 30 min before stimulation with ET-1 (100 nM) for 3 h, subcellular localization of NFATc4 was determined using immunofluorescence staining. Cells were stained with a primary antibody for NFATc4 and an Alexa Fluor 488-conjugated secondary antibody. Cell nuclei were stained with DAPI. (**d**) PASMCs were pre-treated with or without CsA (10 μM) for 30 min before stimulation with ET-1 (100 nM) for 3 h, protein level of NFATc4 in the cytoplasmic and nuclear fractions were determined with immunoblotting. Lamin B and β-actin served as loading controls for the nuclear and cytoplasmic fractions, respectively (n = 4 each group). (**e**) PASMCs were pre-treated with or without CsA (10 μM) for 30 min before stimulation with ET-1 (100 nM) for 24 h, TRPC6 mRNA level was determined using qRT-PCR, β-actin served as an internal control (n = 4 each group). (**f**) PASMCs were pre-treated with or without CsA (10 μM) for 30 min before stimulation with ET-1 (100 nM) for 48 h, TRPC6 protein level was determined using immunoblotting, β-actin served as a loading control (n = 4 each group). *P < 0.05 versus control, ^#^P < 0.05 versus ET-1-treated cells.

Studies have shown that calcineurin/NFAT signaling plays an important role in the regulation of TRPC6 expression in cardiomyocytes and podocytes^25, 26^. To investigate whether activation of calcineurin/NFATc4 signaling pathway mediates the effect of ET-1 on TRPC6 up-regulation, PASMCs were prior incubated with calcineurin inhibitor cyclosporine A (CsA) (10 μM) for 30 min, followed by stimulation with 100 nM ET-1, and then the calcineurin activity, phosphorylation and distribution of NFATc4, and TRPC6 expression were measured. The results show that the presence of CsA significantly suppressed ET-1-induced calcineurin activation, which dropped from 2.13-fold over control in ET-1-treated cells to 1.09-fold over control in CsA and ET-1 co-treated cells (P < 0.05) (Fig. 3a). Figure 3b shows that reduction of NFATc4 phosphorylation was increased from 0.39-fold over control in ET-1-treated cells to 0.79-fold over control in CsA and ET-1 co-treated cells (P < 0.05), and CsA had no effect on the total protein level of NFATc4. Immunofluorescence staining indicated that pre-incubation of cells with CsA suppressed NFATc4 translocation to the nucleus induced by ET-1 (Fig. 3c). The protein level of NFATc4 in cell nucleus declined from 2.02-fold over control in ET-1-treated cells to 1.33-fold over control in CsA and ET-1 co-treated cells (P < 0.05) (Fig. 3d). As shown in Fig. 3e and f, the presence of CsA dramatically reduced ET-1-induced TRPC6 up-regulation, TRPC6 mRNA and protein levels declined from 3.08-fold and 2.31-fold over control in ET-1-treated cells to 1.21-fold and 1.25-fold over control in CsA and ET-1 co-treated cells, respectively (both P < 0.05).

**Figure 4:**
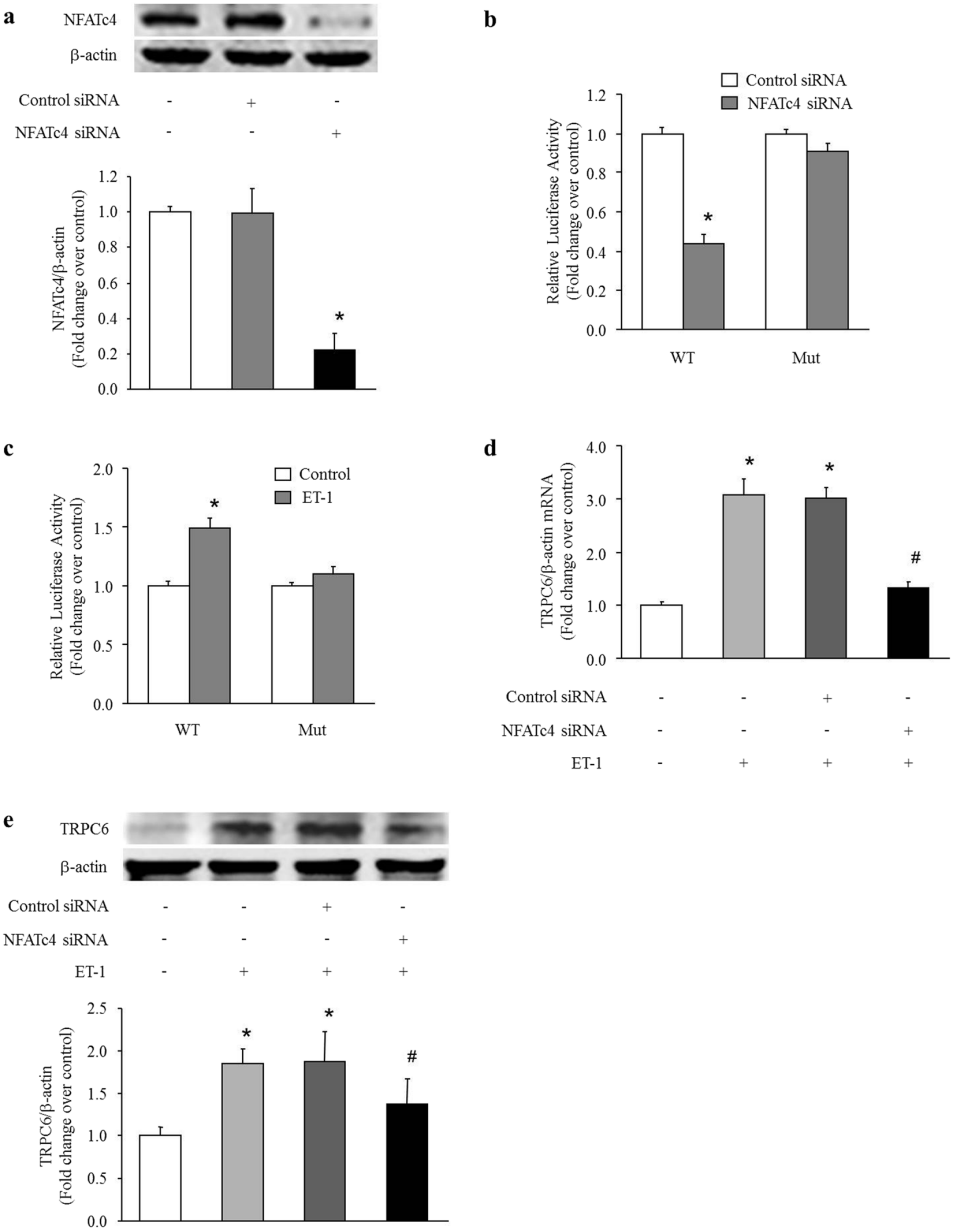
NFATc4 mediates the effects of ET-1 on TRPC6 expression in PASMCs. (**a**) PASMCs were transfected with NFATc4 sequence-specific siRNA and non-targeting siRNA. NFATc4 protein level was determined using immunoblotting, β-actin served as a loading control (n = 4 each group). (**b**) PASMCs were prior transfected with NFATc4-specific or non-targeting siRNA for 24 h, then pGL3-TRPC6-promoter-WT or pGL3-TRPC6-promoter-Mut reporter vector and pRL-TK vector were co-transfected into PASMCs, and luciferase assay was performed 48 h after siRNA transfection (n = 4 each group). (**c**) pGL3-TRPC6-promoter-WT or pGL3-TRPC6-promoter-Mut reporter vector and pRL-TK vector were co-transfected into PASMCs followed by treatment with 100 nM ET-1 for 24 h, then luciferase assay was performed (n = 4 each group). (**d**) PASMCs were prior transfected with NFATc4-specific or non-targeting siRNA for 24 h, and then treated with ET-1 (100 nM) for 24 h, TRPC6 mRNA level was determined using qRT-PCR, β-actin served as an internal control (n = 4 each group).(**e**) PASMCs were prior transfected with NFATc4-specific or non-targeting siRNA for 24 h, and then treated with ET-1 (100 nM) for 48 h, TRPC6 protein level was determined using immunoblotting, β-actin served as a loading control (n = 4 each group). *P < 0.05 versus control, ^#^P < 0.05 versus ET-1-treated cells.

To examine the specific role of NFATc4 in ET-1-induced TRPC6 up-regulation in PASMCs, siRNA-mediated NFATc4 knockdown was performed. Figure 4a shows that transfection of NFATc4 siRNA reduced NFATc4 protein level by 78% (P < 0.05 versus control), while transfection of non-targeting control siRNA did not affect the NFATc4 protein expression. In order to determine whether NFATc4 directly binds to the promoter of TRPC6 and regulates its expression in PASMCs, we performed luciferase assay in PASMCs transfected with pGL3-TRPC6-promoter-WT or pGL3-TRPC6-promoter-Mut luciferase reporter plasmids. As shown in Fig. 4b, co-transfection of NFATc4 siRNA and pGL3-TRPC6-promoter-WT significantly suppressed the luciferase activity in PASMCs, which declined to 0.44-fold compared with cells co-transfected with control siRNA and pGL3-TRPC6-promoter-WT (P < 0.05). In contrast, co-transfection of NFATc4 siRNA or control siRNA with pGL3-TRPC6-promoter-Mut did not affect the luciferase activity in PASMCs. These results suggest that TRPC6 is a direct target of NFATc4 in PASMCs. Figure 4c shows that ET-1 dramatically increased the luciferase activity in PASMCs transfected with pGL3-TRPC6-promoter-WT, which raised to 1.49-fold compared with control cells transfected with pGL3-TRPC6-promoter-WT (P < 0.05). However, treatment of cells with ET-1 did not affect the luciferase activity in PASMCs transfected with pGL3-TRPC6-promoter-Mut. In addition, knockdown of NFATc4 notably reduced TRPC6 expression induced by ET-1 in PASMCs, TRPC6 mRNA and protein levels were decreased from 3.06-fold and 1.84-fold over control in ET-1-treated cells to 1.32-fold and 1.38-fold over control in ET-1 treated cells lacking NFATc4, respectively (both P < 0.05) (Fig. 4d and e). These results suggest that activation of calcineurin/NFATc4 signaling pathway is responsible for ET-1-induced TRPC6 up-regulation in PASMCs.

### Inhibition of PDE5 suppresses the effects of ET-1 on TRPC6 expression in PASMCs

**Figure 5:**
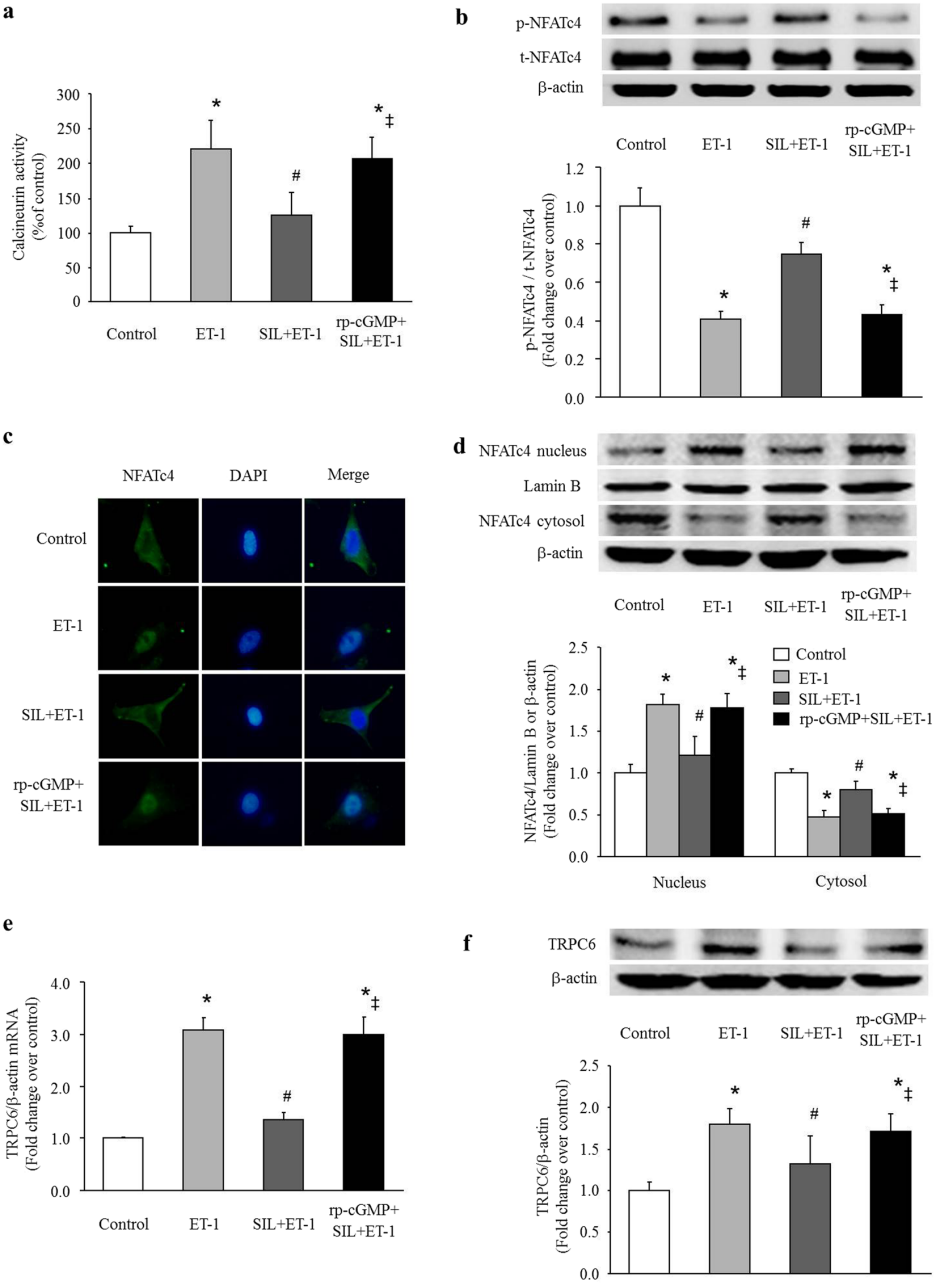
Sildenafil (SIL) inhibits the effects of ET-1 on calcineurin/NFATc4 activation and TRPC6 expression in PASMCs. (**a**) PASMCs were stimulated with ET-1 (100 nM) for 30 min with or without pre-treatment of cells with sildenafil (1 μM for 30 min) or sildenafil plus Rp-8Br-cGMPs (25 μM), calcineurin activity was determined using a calcineurin assay kit (n = 4 each group). (**b**) PASMCs were stimulated with ET-1 (100 nM) for 3 h with or without pre-treatment of cells with sildenafil (1 μM for 30 min) or sildenafil plus Rp-8Br-cGMPs (25 μM), phosphorylation of NFATc4 was measured by immunoblotting, β-actin served as a loading control (n = 4 each group). (**c**) PASMCs were stimulated with ET-1 (100 nM) for 3 h with or without pre-treatment of cells with sildenafil (1 μM for 30 min) or sildenafil plus Rp-8Br-cGMPs (25 μM), subcellular localization of NFATc4 was determined using immunofluorescence staining. Cells were stained with a primary antibody for NFATc4 and an Alexa Fluor 488-conjugated secondary antibody. Cell nuclei were stained with DAPI. (**d**) PASMCs were stimulated with ET-1 (100 nM) for 3 h with or without pre-treatment of cells with sildenafil (1 μM for 30 min) or sildenafil plus Rp-8Br-cGMPs (25 μM), protein level of NFATc4 in the cytoplasmic and nuclear fractions were determined with immunoblotting. Lamin B and β-actin served as loading controls for the nuclear and cytoplasmic fractions, respectively (n = 4 each group). (**e**) PASMCs were stimulated with ET-1 (100 nM) for 24 h with or without pre-treatment of cells with sildenafil (1 μM for 30 min) or sildenafil plus Rp-8Br-cGMPs (25 μM), TRPC6 mRNA level was determined using qRT-PCR, β-actin served as an internal control (n = 4 each group). (**f**) PASMCs were stimulated with ET-1 (100 nM) for 48 h with or without pre-treatment of cells with sildenafil (1 μM for 30 min) or sildenafil plus Rp-8Br-cGMPs (25 μM), TRPC6 protein level was determined using immunoblotting, β-actin served as a loading control (n = 4 each group). *P < 0.05 versus control, ^#^P < 0.05 versus ET-1-treated cells, ^‡^P < 0.05 versus sildenafil and ET-1 co-treated cells.

To determine whether inhibition of PDE5 suppresses TRPC6 expression induced by ET-1 in PASMCs by regulating the calcineurin/NFATc4 signaling pathway, cells were pre-treated with specific PDE5 inhibitor sildenafil (1 μM) for 30 min, and then were stimulated with ET-1 (100 nM). As shown in Fig. 5a, sildenafil dramatically inhibited ET-1-induced calcineurin activation, calcineurin activity declined from 2.21-fold over control in ET-1-treated cells to 1.25-fold over control in sildenafil and ET-1 co-treated cells (P < 0.05). Figure 5b shows that treatment of cells with sildenafil suppressed ET-1-induced dephosphorylation of NFATc4 without changing the total amount of NFATc4, phosphorylation level of NFATc4 was elevated from 0.41-fold over control in ET-1-treated cells to 0.75-fold over control in sildenafil and ET-1 co-treated cells (P < 0.05). Figure 5c shows that sildenafil reduced the increase of fluorescence-labeled NFATc4 in cell nucleus triggered by ET-1. As shown in Fig. 5d, NFATc4 protein level in cell nucleus declined from 1.82-fold over control in ET-1-treated cells to 1.21-fold over control in sildenafil and ET-1 co-treated cells (P < 0.05). Furthermore, the presence of sildenafil dramatically reduced ET-1-induced TRPC6 expression (Fig. 5e and f), TRPC6 mRNA and protein levels dropped from 3.08-fold and 1.79-fold over control in ET-1-treated cells to 1.35-fold and 1.32-fold over control in sildenafil and ET-1 co-treated cells, respectively (both P < 0.05). These results suggest that inhibition of PDE5 suppresses TRPC6 expression induced by ET-1 in PASMCs by modulating the calcineurin/NFATc4 signaling pathway.

To examine whether the above effects of sildenafil on PASMCs was particularly mediated by cyclic guanosine monophosphate (cGMP)-dependent protein kinase (PKG), cells were pre-incubated simultaneously with PKG inhibitor Rp-8Br-cGMPs (25 μM) and sildenafil (1 μM) for 30 min before ET-1 (100 nM) stimulation. The results showed that Rp-8Br-cGMPs abolished the inhibitory effects of sildenafil on calcineurin/NFATc4 activation and TRPC6 up-regulation induced by ET-1 in PASMCs (Fig. 5a–f), the calcineurin activity increased to 2.06-fold over control, the phosphorylation level of NFATc4 declined to 0.43-fold over control (total amount of NFATc4 was not affected), fluorescence-labeled NFATc4 translocated to cell nucleus and the NFATc4 protein level in cell nucleus raised to 1.78-fold over control, TRPC6 mRNA and protein levels increased to 2.99-fold and 1.71-fold over control again, respectively (all P < 0.05 versus sildenafil and ET-1-treated cells). In addition, calcineurin activity, phosphorylation of NFATc4, NFATc4 protein level in cell nucleus, TRPC6 mRNA and protein levels did not show any difference between the ET-1-treated cells and the cells exposure to Rp-8Br-cGMPs, sildenafil and ET-1 (all P > 0.05). These data indicate that inhibition of PDE5 suppresses calcineurin/NFATc4- mediated TRPC6 expression in a cGMP-PKG-dependent manner in PASMCs.

## Discussion

We have shown in this study that ET-1 increases the expression of TRPC6 in PASMCs, this effect is coupled to the particular activation of calcineurin and subsequent NFATc4 translocation to cell nucleus. Furthermore, we have found that inhibition of PDE5 by sildenafil suppresses ET-1-induced activation of calcineurin/NFATc4 signaling pathway and consequent TRPC6 up-regulation in a cGMP-PKG-dependent manner in PASMCs.

TRPC6 is a member of the TRPC subfamily of Ca^2+^-permeable non-selective cation channels expressed in a number of tissues and cells including brain, kidney, lung, smooth muscle cells, lymphocytes, and platelets^28–30^. TRPC6 has been shown to be closely involved in the pathogenesis of several diseases, including PH, cardiac hypertrophy, focal segmental glomerulosclerosis and fibrotic stenosis in Crohn’s disease^9, 25, 26, 31^. In PH, TRPC6 has been shown to play an important role in the proliferation of PASMCs^11^. Increased TRPC6 protein expression has been observed in PASMCs from patients with IPAH^11^. And down-regulation of TRPC6 has been shown to inhibit the increase in intracellular Ca^2+^ level and efficiently attenuate PASMCs proliferation^11, 12^. ET-1, a potent endogenous vasoconstrictor and smooth muscle cell mitogen that is important in PH pathogenesis, has been identified to enhance the expression of TRPC6 in PASMCs. In addition, up-regulation of TRPC6 has been shown to be involved in ET-1-induced PASMCs proliferation^13^. However, mechanisms underlying ET-1 induction of TRPC6 expression in PASMCs are still unknown.

Calcineurin is a Ca^2+^ and calmodulin dependent serine/threonine protein phosphatase that has been implicated in various signaling pathways^32^. Activated calcineurin, in turn, dephosphorylates NFATs, which then translocate from the cytosol to the nucleus and initiate transactivation of the target genes associated with cellular proliferation, differentiation, inflammation and angiogenesis, including TRPC6^33, 34^. The NFAT family consists of at least five members: NFATc1-c4 and NFAT5. NFATc4 isoform is the prominent isoform in PASMCs^35, 36^. A wide variety of stimuli including ET-1 can raise intracellular Ca^2+^ level and activate calcineurin/NFAT signaling pathway^18, 37^. Activation of calcineurin/NFAT signaling pathway has been shown to be associated with the development of PH through induction of PASMCs proliferation and pulmonary vascular remodeling^18, 38^. A study by Nijenhuis *et al*. has shown that activation of calcineurin/NFAT mediates the up-regulation of TRPC6 expression induced by Angiotensin II in podocytes, and overexpression of active NFATc1 in mice podocytes increases TRPC6 expression and induces severe proteinuria^26^. Activation of calcineurin/NFATc4 signaling pathway has also been shown to up-regulate TRPC6 expression in cardiomyocytes to contribute to the development of cardiac hypertrophy^25^. In this study, we further showed that calcineurin/NFATc4 signaling pathway also mediated ET-1-induced TRPC6 up-regulation in PASMCs. Targeting calcineurin/NFATc4 pathway or luciferase reporter assay indicated that TRPC6 lay down-stream of calcineurin/NFATc4 and was directly regulated by NFATc4 in PASMCs.

PDE5 is a member of the PDE superfamily abundantly expressed in lung tissue. This phosphodiesterase specifically hydrolyzes cGMP. As a second messenger, cGMP can activate its down-stream effectors such as PKG, cGMP-regulated phosphodiesterases and cGMP-gated channels, which in turn modulate the activities of a number of proteins that are involved in various cellular processes^39^. PDE5 expression and activity are increased in PH^40^ and may lead to vasoconstriction and proliferation of PASMCs by degradation of cGMP^41, 42^. Sildenafil, a selective PDE5 inhibitor widely used as an effective treatment for clinical PH, has been shown to inhibit PASMCs proliferation by several mechanisms^43–46^. A recent study has found that sildenafil may inhibit chronic hypoxia-induced increase of basal intracellular Ca^2+^ concentration in PASMCs by down-regulation of TRPC1 and TRPC6 expression, thereby contributing to decreased vascular tone of pulmonary arteries during the development of CHPH^23^. In this study, we further indicated that inhibition of PDE5 by sildenafil suppressed ET-1-induced activation of calcineurin/NFATc4 signaling pathway and consequent TRPC6 up-regulation. All these findings suggest that TRPC6 is another target of PDE5 inhibitor in PASMCs. In addition, we found that the inhibitory effects of sildenafil on ET-1-induced calcineurin/NFATc4 activation and TRPC6 up-regulation were abolished by PKG inhibitor, indicating that the above effects of sildenafil on PASMCs are largely depend on cGMP-PKG pathway. Inhibition of PDE5 has been shown to be an important strategy for the clinical treatment of PH, our study provides novel mechanisms underlying the protective effects of PDE5 inhibition in the treatment of PH.

In conclusion, we have identified that ET-1 stimulates TRPC6 expression in PASMCs through activation of calcineurin/NFATc4 signaling pathway, and inhibition of PDE5 suppresses calcineurin/NFATc4- mediated TRPC6 expression in a cGMP-PKG-dependent manner in PASMCs.

## Materials and Methods

### Cell preparation and culture

Primary PASMCs were obtained from pulmonary arteries of male Sprague-Dawley rats (70–80 g) according to the method described previously^19^. All animal care and experiments were performed in accordance with the Guide for the Care and Use of Laboratory Animals of Xi’an Jiaotong University Animal Experiment Center. All protocols used in this study were approved by the Laboratory Animal Care Committee of Xi’an Jiaotong University. In brief, pulmonary arteries were rapidly isolated from euthanized rats by CO_2_ overdose. The adventitia was gently stripped off with forceps and the endothelium was carefully scraped off with elbow tweezers. The remaining smooth muscle layer was cut into small pieces (1 mm × 1 mm) and transferred into a culture flask in Dulbecco’s Modified Eagle Medium (DMEM, Gibco, Grand Isle, NY, USA) supplemented with 10% fetal bovine serum (FBS, Sijiqing, Hangzhou, China) and antibiotics (100 U/ml penicillin and 100 μg/ml streptomycin), and incubated at 37 °C in a humidified 5% CO_2_ incubator. Cells were passaged at 80% confluence using 0.25% trypsin (Invitrogen, Carlsbad, CA, USA). Cells between passages 4–6 were used for further experiments. The purity of PASMCs was confirmed by immunostaining for α-smooth muscle actin (α-SMA) as previously described^19^. Fluorescence microscope images indicated that more than 90% of cells are smooth muscle cells (data not shown here). Cells were serum-starved (1% FBS in DMEM) overnight before each experiment. ET-1 (Enzo Life Sciences, Farmingdale, NY, USA) was used to stimulate PASMCs. Sildenafil (Pfizer, NY, USA) was used to inhibit PDE5. Rp-8Br-cGMPs (guanosine 3′,5′-cyclic monophosphorothioate, 8-bromo-, Rp-isomer, sodium salt) (Abcam, Cambridge, UK) was used to inhibit PKG.

### Quantitative real-time polymerase chain reaction (qRT-PCR)

Total RNA was extracted from PASMCs using TRIzol reagent (Invitrogen) following the manufacturer’s instructions. cDNA was synthesized using RevertAid First Strand cDNA Synthesis Kit (Thermo Scientific, Logan, UT, USA). The cDNA synthesized was used to perform quantitative PCR on an IQ™5 Multicolor Real-Time PCR Detection System (Bio-Rad, Richmond, CA, USA) using the SYBR Green Real-time PCR Master (TaKaRa, Tokyo, Japan). Primers for TRPC6 and β-actin were purchased from Sangon Biotech (Shanghai, China). Primers were as follows: TRPC6, Forward, 5′-GGTGCGGAAGATGCTAGAAG-3′, Reverse, 5′-AATTTCCAGGTGCTCATTGG-3′; β-actin, Forward, 5′-ACGGTCAGGTCATCACTATCGGCAATG-3′, Reverse, 5′-ACAGCACTGTGTTGGCATAGAGGTCTT-3′. Amplification was performed at 95 °C for 30 s, followed by 40 cycles of 95 °C for 5 s and 58 °C for 30 s and 72 °C for 30 s. The fold increase relative to control samples was determined by 2^−ΔΔCt^ method. The expression of TRPC6 was normalized to β-actin.

### Transfection of siRNA

PASMCs were seeded in 6-well plates and cultured till reaching 30–50% confluence. Then cells were transfected with 100 nM NFATc4 siRNA, or non-targeting control siRNA (GenePharma, Shanghai, China) using Lipofectamine™ 2000 Reagent (Invitrogen) according to the manufacturer’s protocols. Briefly, siRNA and Lipofectamine were diluted separately in serum-free DMEM, and incubated for 5 min at room temperature. Next, the diluted siRNA was gently mixed with the diluted Lipofectamine and incubated for 20 min at room temperature. Then, the complex of siRNA and Lipofectamine was added into cells. The cells were incubated in serum-free DMEM containing the siRNA and Lipofectamine complex for 6 h, after which the medium was replaced with fresh DMEM containing 10% FBS. Effect of siRNA transfection was analyzed using immunoblotting following a further 48 h culture in a 37 °C, 5% CO_2_ humidified incubator.

### Luciferase reporter assay

One potential NFATc4 binding site within TRPC6 promoter (AGAATTTTCC) was identified in silico analysis (PROMO using version 8.3 of TRANSFAC), which was used to create luciferase reporters. The wild-type TRPC6 promoter (WT) (AGAATTTTCC) and the mutant TRPC6 promoter (Mut) (TCATTAATCC) were established and cloned into the pGL3-basic vector (Promega, Madison, WI, USA) by Sangon Biotech (Shanghai, China), named as pGL3-TRPC6-promoter-WT and pGL3-TRPC6-promoter-Mut, respectively. PASMCs were seeded into 24-well plates and transfected with NFATc4 siRNA or control siRNA. 24 h after siRNA transfection, the cells were co-transfected with pGL3-TRPC6-promoter-WT or pGL3-TRPC6-promoter-Mut reporter vector and renilla luciferase-expressing vector pRL-TK (Promega) using Lipofectamine™ 2000 (Invitrogen). After another 24 h, cells were harvested and firefly and renilla luciferase activities were measured using the dual-luciferase reporter assay system (Promega). Luciferase activity of pRL-TK was used as an internal control to normalize transfection and harvest efficiencies. Transfections were performed in triplicate and repeated four times in separate experiments.

### Preparation of cytoplasmic and nuclear extracts and whole cell lysates

Whole cell lysates were extracted according to the method described as follows: Cells were gently washed twice in ice-cold PBS and lysed in RIPA lysis buffer containing 50 mM Tris-HCl (pH 7.4), 1% NP-40, 0.1% sodium dodecyl sulfate (SDS), 150 mM NaCl, 0.5% sodium deoxycholate, 1 mM EDTA, 1 mM phenylmethanesulfonyl fluoride, 1 mM Na_3_VO_4_, 1 mM NaF and proteinase inhibitors. Lysates were then sonicated and centrifuged at 12,000 g for 10 min at 4 °C, and the supernatants were collected as whole cell lysates. The isolation of cytoplasmic and nuclear extracts was performed using a cytoplasmic and nuclear protein extraction kit (Heart Biotech, Xi’an, China). Briefly, cells were washed twice with ice-cold PBS, scraped and pelleted through centrifugation (800 g for 5 min at 4 °C). The pellets were then dissolved in ice-cold cytoplasmic extraction reagent, incubated on ice for 15 min and vortexed at high speed for 10 sec. Samples were then centrifuged at 12,000 g for 10 min at 4 °C and the supernatant was collected as cytoplasmic fractions. The pelleted nuclei were re-suspended in a nuclear extraction buffer and incubated on ice for 30 min with vortexing at 5 min intervals. After centrifugation at 12,000 g for 10 min at 4 °C, the supernatant was collected as nuclear fraction. The concentration of each extract was measured using a BCA protein assay kit (Pierce Biotechnology, Rockford, IL, USA).

### Western blot analysis

Proteins were separated on 6–10% SDS-PAGE gel and transferred onto nitrocellulose membranes (Bio-Rad). After blocking for 1 h at room temperature with 5% nonfat dry milk or 3% BSA in PBST (PBS containing 0.1% Tween-20), the membranes were incubated overnight at 4 °C with primary antibodies against TRPC6 (1:500 dilution; sc-19196, Santa Cruz Biotechnology, Santa Cruz, CA, USA), p-NFATc4 (1:200 dilution; sc-135771, Santa Cruz), NFATc4 (1:500 dilution; BS1762, Bioworld Technology, St. Louis Park, MN, USA), Lamin B (1:1,000 dilution; 12987-1-AP, Proteintech, Chicago, IL, USA), and β-actin (1:2,000 dilution; sc-47778, Santa Cruz). Membranes were then washed three times in PBST for 15 min, followed by incubation at room temperature for 2 h with horseradish peroxidase (HRP)-conjugated rabbit anti-goat secondary antibody (1:5,000 dilution; SA00001-4, Proteintech), goat anti-rabbit secondary antibody (1:5,000 dilution; A0545, Sigma-Aldrich, St. Louis, MO, USA) or goat anti-mouse secondary antibody (1:5,000 dilution; SA00001-1, Proteintech). Protein bands were visualized using the enhanced chemiluminescence (ECL) kit (Thermo Scientific). Images were digitally captured using a ChemiDoc XRS System (Bio-Rad). The band densities were quantified using Quantity One software (Bio-Rad).

### Immunofluorescence staining

Cells were fixed with 4% paraformaldehyde in PBS for 20 min at room temperature. After washing three times with PBS (5 min each), cells were permeabilized with 0.3% Triton X-100 in PBS for 10 min, and then washed a further three times with PBS (5 min each). Permeabilized cells were blocked with 5% BSA in PBS for 30 min at room temperature, and then incubated overnight with the primary antibody against NFATc4 (1:50 dilution; BS1762, Bioworld Technology) at 4 °C. The cells were washed three times with PBS (5 min each) and then incubated in the dark for 1 h at room temperature with Alexa Fluor 488-conjugated goat anti-rabbit secondary antibody (1:200 dilution; ab150077, Abcam). Finally, nuclei of cells were stained with 4′,6-diamidino-2-phenylindole (DAPI) (Sigma-Aldrich). Stained cells were observed and photographed using an inverted fluorescence microscope (Nikon, Japan).

### Calcineurin activity assay

PASMCs from different groups were lysed in 400 μl lysis buffer composed of 50 mM Tris-HCl, 0.1 mM EDTA, 0.1 mM EGTA, 0.2% NP-40, 1 mM DTT and protease inhibitor cocktail. The supernatant was collected for assay by centrifugation (4 °C, 13,000 rpm, 10 min). Protein concentration was measured using a BCA protein assay kit (Pierce). Then calcineurin phosphatase activity was measured using a calcineurin assay kit (Nanjing Jiancheng Bioengineering Institute, Nanjing, China) according to the manufacturer’s instruction.

### Measurement of [Ca^2+^]i

Intracellular Ca^2+^ concentration ([Ca^2+^]i) was measured using the membrane-permeable Ca^2+^-sensitive fluorescent dye Fluo-3 AM (Beyotime Biotechnology, Jiangsu, China). Briefly, PASMCs were loaded with 5 μM Fluo-3 AM for 30 min at 37 °C in Hanks’ balanced salt solution (HBSS) (137 mM NaCl, 0.3 mM Na_2_HPO_4_, 4.2 mM NaHCO_3_, 1.3 mM CaCl_2_, 0.5 mM MgCl_2_, 0.6 mM MgSO_4_, 5.4 mM KCl, 0.4 mM KH_2_PO_4_, 5.6 mM glucose, pH 7.4). Cells were then washed with HBSS and rested for 20 min to remove extracellular Fluo-3 AM and to allow for intracellular dye cleavage. For fluorescence measurements, images were captured every 5 sec by excitation at 488 nm and emission light of 525–530 nm using a laser scanning confocal microscopy (Nikon).

### Statistical analysis

Data are presented as mean ± standard deviation (S.D.). Differences among groups were analyzed using one-way analysis of variance followed by Tukey post hoc test. P < 0.05 was considered statistically significant.
